# Supplementary material for: Organismal metabolism regulates the expansion of oncogenic PIK3CA mutant clones in normal esophagus
Source: Nat Genet. 2024 Aug 21;56(10):2144–57. doi: 10.1038/s41588-024-01891-8 (PMC11525199; doi:10.1038/s41588-024-01891-8)
Supplement: Supplementary file 1 — Supplementary Note: Additional text, Methods and Figures. [file 41588_2024_1891_MOESM1_ESM.pdf]

nature genetics

Supplementary information https://doi.org/10.1038/s41588-024-01891-8

Organismal metabolism regulates the expansion of oncogenic *PIK3CA* mutant clones in normal esophagus

In the format provided by the authors and unedited

Supplementary Note

Contents

Page

2-3 Metabolic changes downstream of Pik3ca^{H1047R} and competitive fitness

4-10 Modelling wild type and mutant cell behavior

11-16 Additional Methods

11 *Pik3ca*$^{fl\text{-}H1047R\text{-}T2A\text{-}EYFP\text{-}NLS/wt}$ mouse generation

11 Validation of *Pik3ca*^{H1047R} mutant construct

12 Immune capillary electrophoresis

13 Western blotting

13-15 CRISPR/*Cas9* screening

15 Real-time qPCR

15-16 RNA isolation and RNA sequencing

17 Supplementary Figure 1

18 Supplementary Figure 2

19 Supplementary Figure 3

20 Supplementary References

Metabolic changes downstream of Pik3ca^{H1047R} and competitive fitness of mutant cells

Here we detail additional investigations of the downstream metabolic effects of expression of the Pik3ca^{H1047R} mutant and their impact on competitive fitness.

We investigated whether the metabolic switch observed in *Pik3ca* mutant cells impacted their competitive fitness or was just a consequence of the increased HIF1α signaling. The connection between glycolysis and cell competition is controversial. Metabolic reprogramming towards glycolysis alters cell competitive fitness in Drosophila [1,2]. In mammalian keratinocytes, glycolysis has been linked to cell fate regulation and differentiation suggesting that glycolytic activation may confer a proliferative advantage in the esophagus [3-5]. gRNAs targeting most glycolysis pathway genes were depleted in the screen, including *Ldha*, which is not implicated in mitochondrial glucose oxidation, implying that glycolysis regulates cell fitness (**Extended Data Fig. 5c and Supplementary Fig. 2a**). Intriguingly, however, deletion of the glucose transporter *Glut1* showed the opposite effect. This argues that modulation of glycolysis at different levels may have different effects on competitive fitness. This may be explained by effects on differentiation. Cytosolic glucose has been shown to promote keratinocyte differentiation while activation of downstream glycolysis inhibits keratinocyte differentiation [6,7]. Consistent with a link between glycolysis and mutant cell fitness advantage, mutant cells are slightly more sensitive to the loss of *Ldha*, *Aldoa*, *Eno1* and *Pgam1* than wild-type cells (**Extended Data Fig. 5c and Supplementary Fig. 2a-b**) and similar effects were observed in INS treated WT cells (**Supplementary Fig. 2c**). These results would suggest that the fitness of cells with higher PI3K activity is slightly more dependent on glycolysis.

Is glycolysis the only metabolic pathway responsible for the increased fitness of mutant cells? Surprisingly, removing glucose from culture medium or treating with the glucose uptake inhibitor 2-deoxyglucose did not affect *Pik3ca*$^{H1047R/wt}$ cell fitness advantage in mixed mutant/wild-type cultures (**Extended Data Fig. 8a-b**). Conversely, treating with DCA, which by inhibiting pyruvate dehydrogenase kinase-1 favors mitochondrial glucose oxidation over aerobic glycolysis to lactate, modestly but significantly reduced mutant cell advantage (**Extended Data Fig. 8c-e**) [8-10]. In agreement with the *in vitro* results, DCA treatment *in vivo* for 28 days reduced mutant clone size and increased the proportion of differentiated mutant cells towards the levels observed in wild-type clones (**Extended Data Fig. 8f-i**). A non-parametric two-factor analysis (see methods) revealed that DCA significantly decreased the differences between wild-type and mutant basal clone size distributions (**Extended Data Fig. 8j**). Because of the impact of DCA on multiple organs *in vivo*, it is important to note that its effect on *Pik3ca*$^{H1047R/wt}$ cell fitness could be via the direct targeting of esophageal glycolysis or by reducing insulin blood levels or a combination of both [8-

[10]. These results suggest that glycolysis activation only partially explains the competitive fitness advantage of *Pik3ca*$^{H1047R/wt}$ over wild-type cells.

A metabolic switch to aerobic glycolysis is frequently accompanied by an increased glutaminolysis and lipogenesis, which are regulated by the PI3K/AKT/mTOR pathway [11-13]. The *Pik3ca*^{H1047R} mutation is described to activate *de novo* lipogenesis and glutaminolysis [14,15]. We therefore checked the effect of glutaminolysis and lipogenesis inhibitors on mutant-wild-type cell competition in culture. The glutaminolysis inhibitor CB839 did not significantly affect *Pik3ca*^{H1047R} cell fitness (**Extended Data Fig. 8k**). However, inhibiting either *Fasn* or *Acc*, two of the main steps in lipogenesis pathway, reduced the advantage of mutant over wild-type cells (**Extended Data Fig. 8k**). Treatment with the *Srebp1c* inhibitor Betulin did not significantly alter mutant cell behavior, arguing that the changes in *Pik3ca* mutant cells are not mediated by transcriptional changes (**Extended Data Fig. 8k**). Consistent with this, mutant cells had similar expression of most *Srebp1c* lipogenesis-related targets (*Acly*, *Acaca*, *Acacb*, *Fasn* or *Scd1*), only the mitochondrial citrate exporter (*Slc25a1*) and *Scd2* showed increased expression in mutant cells (**Extended Data Fig. 6l**).

In summary, *Pik3ca* mutant cells show metabolic rewiring with increased HIF1α signaling and glycolysis. This is only partially responsible for their competitive advantage. The demonstration that lipogenesis inhibition reduces mutant cell fitness suggests that multiple metabolic pathways downstream PI3K pathway promote mutant cell competitiveness. Further work using more specific inhibitors of ACC, lipid deprivation or fatty acid treatments will be needed to confirm the importance of *de novo* lipogenesis in esophageal epithelium cell fate and *Pik3ca*$^{H1047R/wt}$ cell mutant advantage.

Modelling wild type and mutant cell behavior

This section describes the quantitative methods and modelling of mutant progenitor cell behavior **Section 1** covers the analysis of wild-type progenitor behavior. In **Section 2** we extend the methodology to test *Pik3ca* mutant progenitor dynamics. In **Section 3** we analyze cell dynamics in the suprabasal compartment for model validation.

1. Wild-type progenitor cell dynamics

The murine esophageal epithelium consists of layers of keratinocytes maintained by a single type of proliferative cells [16,17]. Progenitor (P-) cells reside in the deepest, basal layer where they divide regularly at a rate λ. The outcome of a given progenitor division is stochastic: with a certain probability it results in two proliferating daughter cells retaining the proliferative capacity (P+P) or two post-mitotic, differentiating cells (D+D), the remaining divisions yielding an asymmetric outcome (P+D). Upon differentiation, D-cells stratify (at rate Γ) into the upper, suprabasal layers (transiting to S-cells), being ultimately shed into the lumen (at rate μ). This scenario is summarized by the single-progenitor (SP) model (**Fig. 3i**):

$$P \xrightarrow{\lambda} \begin{cases} P+P & \text{Prob. } r \\ P+D & \text{Prob. } 1-2r \\ D+D & \text{Prob. } r \end{cases}$$

$$D \xrightarrow{\Gamma} S \qquad (1)$$

$$S \xrightarrow{\mu} \emptyset$$

While the outcome of individual divisions is unpredictable, overall, the likelihood of the symmetric PP and DD division outcomes is balanced in adult wild-type mice (setting the same probability, *r*). This ensures that on average half the progenitor cells go on to divide and half differentiate, so that the tissue remains homeostatic.

Under homeostasis, one can assume the proportion of proliferative basal cells, ρ, remains constant, and overall, the net rate at which cells are generated in the basal compartment is compensated by cell stratification and cell loss by shedding (**Extended Data Fig. 3h and i**). Then, the following relationships between the parameters can be established: $\Gamma = \lambda\rho/(1-\rho)$, and $\mu = \lambda\rho(1-h)/h$, where h is the proportion of suprabasal cells relative to total cells. Alternatively, one can set $\mu = \lambda\rho/m$, if we define *m* as the global ratio of suprabasal-to-basal cell populations.

Our lineage tracing data from *Cre-RYFP* (wild-type) mice are consistent with key dynamical features of the SP model with balanced fates, in agreement with our more extensive work carried out previously using the same mouse strain and others [16-18]. First, persisting clones (i.e. those retaining at least one basal cell) show ever increasing sizes during the duration of the experiment. In particular, the average number of basal cells per persisting clone follows a linear growth over time (**Fig. 3b**). Second, clones become increasingly heterogeneous in size, both in terms of the number of basal cells and total (basal + 1st suprabasal) cells per clone, the distributions adopting a scaling behavior at late time points (**Fig. 3d**). These are hallmarks of neutral clone competition, where some clones grow by chance at the expense of others that shrink, lose basal attachment and get ultimately extinct by shedding (**Fig. 3h**).

In order to validate the dynamics of the wild-type progenitor cells in our particular experimental setup, we proceeded to determine the values for the unknown SP-model parameters by fitting the experimental basal clone size distributions at the different time points (suprabasal cell numbers are not required for this since proliferative cells are confined to the basal compartment). For the division rate, we took as a prior the average value for λ and the distribution of cell-cycle time periods t_{cc} inferred by H2B-GFP dilution chase experiments in [17], i.e. $<\lambda>$ = 2.9 week^{-1} and $t_{cc} \sim \tau_R + \mathrm{Gam}(\kappa, \vartheta)$, where τ_R = 0.5 day^{-1} (refractory period) and Gam refers to a Gamma distribution with κ = 8 and $\vartheta = (1 - \tau_R < \lambda >)/(\kappa < \lambda >)$. Notice that the election of this realistic description of the division rate will condition the modelling implementation as this cannot rely on Poisson processes that assume independent, underlying exponential events (see below). A maximum likelihood estimation (MLE) approach was then followed to infer the values of the two other remaining parameters, *r* and ρ (which sets the value of Γ in homeostasis), as explained below.

We performed a grid search spanning the range of all possible values for *r* and ρ, and for every parameter set θ we estimated the log-likelihood value $l(\theta; x)$ as in previous work [17,19]:

$$l(\theta; x) = \sum_t \sum_n (x_n(t) * \log p_n(t, \theta)) \qquad (2)$$

$x_n(t)$ is the observed frequency of clones with a certain basal size *n* at time *t*. In turn, $p_n(t, \theta)$ refers to the probability of observing clones of that size at time *t* given the SP model with parameter values θ, and it was obtained by numerical solution (≥ 100,000 simulations) of the Master equation. In particular, in [19], this was computed by implementing a Markov-chain Monte Carlo method (Gillespie's algorithm) [20,21]. Here, we used an exact non-Markovian Monte Carlo analogue developed in [17], which allows to account for the gamma-distributed cell cycle times. For convenience, both experimental and simulated clone sizes were binned in ranges increasing in

powers of two given the large asymmetry of the distributions, so that in practice, n in the equation above stands for clones with a number of basal cells in the range (2^{n-1} + 1, 2^n).

To discard possible biases due to the initial induction of post-mitotic cells (D- or S- cells), only clones with at least two basal cells were considered for the analysis. Also, a small fraction of clones at the latest time point (2 out of 317 clones in the wild-type; 3 out of 563 clones in the mutant) were classified as outliers (i.e. having a number of basal cells >> 2,3 s.d. above the average of that time) and reassigned to the top size range n of non-outlier clones [17,19]. Finally, since there were large differences in our experimental sample size across time points (especially in the wild-type: 1522, 710, 457 and 317 clones quantified at time 10 d, 28 d, 84 d and 168 d, respectively) and these can imprint uneven contributions to $l(\theta; x)$ calculations (**Eq. 2**), here we followed a bootstrapping strategy: We computed $l(\theta; x)$ repeatedly for different sample subsets containing a fixed number of clones X across time points, drawn by random permutation with replacement of the original sampled clones. In this way we ensured even weights from the different time points and more robust parameter estimates by averaging $l(\theta; x)$ across subsamples.

Following this methodology, we obtained the following parameter estimates ($\hat{\theta}_{MLE}$) for the wild-type progenitors (**Fig. 3i and Supplementary Fig. 1a**):

$$\rho = 0.52\ (0.48; 0.63);\ r = 0.08\ (0.07; 0.10) \qquad (3)$$

From these, we derive the stratification rate (recall homeostatic relationships above): Γ = 3.14 (2.68; 4.94) week^{-1}. In parentheses are 95% confidence interval bounds based on likelihood-ratio test (Pearson's χ^2 cutoff with 2 degrees of freedom). Altogether, these parameter values are in good agreement with those found previously from lineage tracing using inducible *Ah-Cre^{ERT}* and *Lrig1-Cre^{ERT}* based mice ({ρ = 0.56 (0.50; 0.89), r = 0.06 (0.04; 0.10)} and {ρ = 0.65 (0.50; 0.96), r = 0.10 (0.07; 0.15)}, respectively) [17]. It follows that using the SP model with the MLE values (**Eq. 3**) we obtained good fits on both the experimental average number of basal cells per clone (**Fig. 3b**) and the distributions of basal clone sizes at the different time points (**Supplementary Fig. 1c**), corroborating the robust dynamics of wild-type progenitors in the esophageal epithelium.

2. *Pik3ca$^{H1047R/wt}$* progenitor cell dynamics

The dynamics of *Pik3ca$^{H1047R/wt}$* mutant progenitor cells clearly differed from wild-type cells as clones showed an accelerated growth over time in both the basal and suprabasal compartments (**Fig. 3b-d**). The percentage of EdU$^+$ basal cells among the induced *Pik3ca$^{H1047R/wt}$* population was similar to wild-type (**Fig. 3e-f**) and comparable to measurements done before in mouse

esophagus [22]. This argues against changes in the rate of mutant cell division λ. Yet, in principle dynamics might still be explained by a SP model with balanced fates if *Pik3ca*$^{H1047R/wt}$ progenitors experienced changes in some of the other parameters (e.g. *r* or Γ). Alternatively, it could be that *Pik3ca*$^{H1047R/wt}$ clone behavior responds to an imbalance in mutant progenitor division outcomes that favors proliferating daughter cells over differentiating progeny (i.e. PP symmetric division outcome being more likely than DD) (**Extended Data Fig. 3i**). This later scenario has been shown to explain the phenotype of some other inducible mutants in squamous epithelium such as *DN-Maml1*, which inhibits the Notch pathway [23], and *Trp53* mutants [19,24]. The distinction between these two possibilities is important since the former involves a neutral scenario where *Pik3ca*$^{H1047R/wt}$ population would exhibit no competitive advantage over wild-type but an exacerbated stochastic behavior (i.e. accelerated clone growth but also decline). By contrast, fate imbalance would introduce a selective advantage, cause a net exponential-like growth of mutant clones and lead to mutant cell colonization of the epithelium.

The supralinear growth observed in the average mutant clone size points towards a progenitor fate imbalance in the *Pik3ca*$^{H1047R/wt}$ population (**Fig. 3b**). Unfortunately, however, the initial clonal induction efficiency was variable between mice and this precluded reliable confirmation of an overall mutant cell colonization. To circumvent this issue, we decided to explore the distributions of the mutant clone sizes (**Fig. 3d**) and extend the maximum likelihood estimation (MLE) approach explained earlier to infer the most likely scenario of mutant progenitor behavior. The following model that allows for a fixed progenitor fate imbalance (Δ) was considered [19,24]:

$$P \xrightarrow{\lambda} \begin{cases} P + P & \text{Prob. } r(1+\Delta) \\ P + D & \text{Prob. } 1-2r \\ D + D & \text{Prob. } r(1-\Delta) \end{cases}$$

$$D \xrightarrow{\Gamma} S \qquad (4)$$

$$S \xrightarrow{\mu} \emptyset$$

Notice that the neutral-case scenario (balanced fates) corresponds with $\Delta = 0$ (see equivalence to **Eq. 1**), and thus, it can be tested as a nested condition from this model. The assumption on the invariant nature of Δ is made for simplification and becomes reasonable given the low (variable) induction and the relatively short time scales in the experiments; different processes could dampen mutant fate imbalance at longer term [19,25].

The deterministic ODE equations that describe the time course of the global cell populations from the model in **Eq. 4** are:

$$\frac{dn_P}{dt} = 2\Delta r \lambda n_P$$

$$\frac{dn_D}{dt} = \lambda n_P - 2\Delta r \lambda n_P - \Gamma n_D \quad (5)$$

$$\frac{dn_S}{dt} = \Gamma n_D - \mu n_S$$

From these, one can infer asymptotical values for ρ and h as t >> $\Delta r \lambda$, so that the following relationships between the parameters can be established:

$$\Gamma = \frac{\lambda\rho - 2\Delta r\lambda}{1-\rho}; \; \mu = \frac{\lambda\rho(1-h) - 2\Delta r\lambda}{h} \quad (6)$$

A parameter grid search was done for different values of $r \in [0, 0.5]$, $\rho \in (0, 1]$ and $\Delta \in [0, 1]$ and stochastic model simulations run to estimate theoretical probabilities that clones develop into different sizes $p_n(t, \theta)$, as set earlier for the wild-type (the mutant average division rate <λ> and cell-cycle time t_{cc} was assumed equal to wild-type). By computing the log-likelihood value $l(\theta; x)$ for every set of parameter values θ on the experimental distributions of the number of basal cells per clone we obtained the following parameter estimates ($\hat{\theta}_{MLE}$ with 95% C.I.) for the *Pik3ca*$^{H1047R/wt}$ progenitors (**Supplementary Fig. 1b**):

$$\rho = 0.63\ (0.49; 0.99);\ r = 0.17\ (0.12; 0.30);\ \Delta = 0.03\ (0.01; 0.06) \quad (7)$$

According to the parameter relationships in **Eq. 6**, Γ = 4.86 (2.72; 285.48) week^{-1}. It follows that independently of the values of r and Γ, a neutral SP model with balanced fates (Δ = 0) was significantly less likely to explain mutant progenitor dynamics than a model with fate imbalance (Δ > 0), implying selection (using most favorable parameters for each model, p = 0.0002, *** by likelihood ratio test). In fact, the best neutral model found could not produce a good fit on the basal clone size distributions nor explain the curvature in the time course of the average number of basal cells per clone in the mutant population. A SP model with fate imbalance (MLE values from **Eq. 7**) gave an excellent fit on these experimental data (**Fig. 3b and Supplementary Fig. 1c**). We conclude that *Pik3ca*$^{H1047R/wt}$ progenitor dynamics are characterized by a small, but existent, statistical bias in fate towards an excess of dividing over differentiating daughters per average cell division. This is accompanied by an overall increased proportion of divisions being symmetric, as reflected by the larger value of r (**Fig. 3i**). Altogether, this would result in individual mutant clones developing into a wider range of possible sizes – more extreme random trajectories – while there is, overall, a relentless colonization of the esophageal epithelium by *Pik3ca*$^{H1047R/wt}$ cell population (**Supplementary Video**).

3. Dynamics of suprabasal cells and total clone behavior

A model of *Pik3ca*$^{H1047R/wt}$ selection based on progenitor fate imbalance sets some predictions on the suprabasal component of mutant clones. Therefore, within the limitations of our experimental model (*Pik3ca*$^{H1047R/wt}$ cells could only be detected up to the 1st suprabasal layer, above which the *Pik3ca* locus expression is too close to the background), we turned to study the dynamics of suprabasal and total cell populations as a validation (at least semi-quantitatively) of our model findings.

First, the introduction of a fate imbalance is expected to decrease the relative proportion of suprabasal cells in the mutant clones. In particular, the global suprabasal-to-total cell ratio *h* in the *Pik3ca*$^{H1047R/wt}$ population would decay with the value of Δr according to the following rational function (**Supplementary Fig. 1d**) (see **Eq. 6**):

$$h = \frac{\Gamma(\lambda - 2\Delta r\lambda)}{\lambda\mu + \Gamma\mu + \lambda(\Gamma - 2\Delta r\lambda)} \qquad (8)$$

Indeed, the average fraction of 1st suprabasal cells in *Pik3ca*$^{H1047R/wt}$ clones was significantly smaller than in wild-type clones across time points (**Fig. 3g**). Intriguingly, the experimental data showed a gradual decline in the proportion of suprabasal cells in both genotypes at the late time points, something a simple model with constant parameters would not capture. This apparent departure from homeostasis might be technical but could be related to epithelial changes as mice age [26]. To achieve a more accurate description of the experimental conditions while retaining the MLE model parameters that suited basal-layer behavior, we thus considered a time-dependent shedding rate $\mu(t)$, which is the only extra adjustable parameter needed to describe suprabasal cell dynamics. In this way, in the simulations of suprabasal and total clone sizes, μ could be adapted to reproduce the variable value of *h* over time (**Eq. 6**).

Independently of whether we implemented time-adjusted values for μ or used a fixed default value given by the experimental average suprabasal-to-total cell ratio *h*, our (zero-parameter) model fits on the distributions of total (basal + 1st suprabasal) cells per clone were adequate over time points (**Fig. 3b and Supplementary Fig.1c**), arguing on the adequacy of the MLE estimates.

Finally, the model predicts differences in the fraction of floating clones (i.e. clones having no basal cells) between wild-type and mutant populations at intermediate time points (**Supplementary Fig. 1e**). These differences persisted regardless of whether only P-cells were considered as initially labelled or all populations were initially induced at even proportions. Floating clones reflect transient clones that have lost the proliferative capacity and are to be lost by terminal differentiation. They are expected to be less abundant if progenitors show a fate imbalance

favoring proliferating over differentiating progeny. This felt in agreement with trends in the experimental data (**Fig. 3h**).

Altogether, mathematical modelling indicates *Pik3ca*$^{H1047R/wt}$ behavior is explained by mutant progenitors showing an increased ability to yield proliferating daughters upon division, which confers this genotype a competitive advantage over wild-type cells.

Code used for model simulations in this study can be found at: https://github.com/gp10/DriverClonALTfate

Additional Methods

Pik3ca$^{fl\text{-}H1047R\text{-}T2A\text{-}EYFP\text{-}NLS/wt}$ mouse generation

Pik3ca$^{fl\text{-}H1047R\text{-}T2A\text{-}EYFP\text{-}NLS/wt}$ knock-in mice were generated by Taconic Biosciences (Hudson, NY). In the targeting vector, exon 20 (including the splice acceptor site of intron 19), the endogenous STOP sequence and the 3'UTR region of the wild-type *Pik3ca* gene were flanked by *loxP* sites. A second *Pik3ca* exon 20 including the *Pik3ca^{H1047R}* mutation was introduced 3' of the distal *loxP* site. Between the last amino acid and the translation termination codon of the duplicated exon 20 the following sequences were inserted: a self-cleaving T2A peptide, enhanced yellow fluorescent protein (EYFP) fused to a Nuclear Localization Signal (NLS), a STOP cassette and the 3′UTR region of the *Pik3ca* gene. Two positive selection markers were also introduced. A Neomycin resistance gene flanked by Frt sites was inserted 3′ of the 5′ *loxP* site before exon 20. A Puromycin resistance gene flanked by F3 sites was placed downstream of the 3′UTR from the duplicated region. An additional polyadenylation signal (hGHpA: human Growth Hormone polyadenylation signal) was inserted between the 3' UTR and the distal *loxP* site to prevent downstream transcription of the mutated *Pik3ca^{H1047R}* exon 20 before Cre recombination. Finally, the vector included a distal thymidine kinase (Tk) gene at the 3' end for negative selection. The targeting vector was generated using BAC clones from the C57BL/6J RPCIB-731 BAC library and transfected into the Taconic Biosciences C57BL/6N Tac ES cell line. Homologous recombinant clones were isolated using double positive (NeoR and PuroR) and negative (Tk) selections. The appropriate insertion of the vector was assessed by PCR. The conditional knock-in allele was obtained after *in vivo* Flp-mediated removal of the selection markers. This allele expresses the wild-type p110α protein. The presence of the hGHpA cassette downstream of the wild-type exon 20 prevents transcription of the mutated H1047R exon 20 and the EYFP before Cre recombination. The constitutive knock-in allele is obtained after Cre-mediated deletion of wild-type exon 20 and the hGHpA, expresses a chimeric transcript harboring the mutated p110α^{H1047R} protein fused to the T2A sequence and the EYFP open reading frame including the NLS. The expected co-translational cleavage at the T2A sequences result in co-expression of the mutated p110α^{H1047R} and EYFP proteins under the control of the endogenous *Pik3ca* promoter.

Validation of *Pik3ca^{H1047R}* mutant construct

DNA that reflects the recombined *Pik3ca$^{H1047R\text{-}YFP}$* allele (*PIK3CA*^{H1047R} fused to self-cleaving peptide P2A and GFP) was chemically synthesized (GenScript USA Inc.). cDNA encoding murine p110α$^{wild\text{-}type}$, p110α^{H1047R} and p110α^{H1047R}-P2A-GFP were amplified by PCR using IMAGE clone (Image ID 40141870/IRCL34 C10 (M13R), Source Bioscience) for wild-type p110α or above cDNA for mutants and subcloned into the pCS2+ expression vector. NIH3T3 cells (ATCC CRL-1658) were transiently transfected with these constructs using Lipofectamine 2000 (Thermo Fisher 11668030) according to manufacturer's instruction. Cells were serum-starved by culture in DMEM containing 0.3% serum for 23 h. Serum-starved NIH3T3 cells were lysed in buffer containing 20 mM Hepes NaOH pH 7.9, 10% Glycerol, 0.4 M NaCl, 0.5% NP-40, 0.2 mM EDTA, 0.01% halt protease and phosphatase inhibitor (ThermoFisher Scientific, cat

#78415). Protein concentrations were measured using standard Bradford protein assays (BioRAD QuickSTART™ Bradford Dye Reagents, cat.no.500-0202). Lysates were mixed with equal amount of 2x loading buffer (100 mM Tris-HCl pH 6.8, 4% SDS, 20% Glycerol, Bromophenol blue and 0.2% β-mercaptoethanol) and boiled at 96°C for 5 min. Samples were loaded onto a 7.5 or 10% of SDS-polyacrylamide gel. Proteins were separated by electrophoresis and transferred onto Immobilon-P membrane (pore size 0.45 µm, Millipore IPVH00010). Membranes were incubated in blocking buffer (5% dried skimmed milk, PBS, 0.1% Tween-20) at room temperature for 1 h and then with primary antibodies diluted 1:1000 in blocking buffer (anti-GFP Abcam Ab290; anti-p110a Abcam Ab152155; anti-P-AKTS473 New England Biolabs 4060S; anti-pAKT308 New England Biolabs 2965S; anti-Akt New England Biolabs 4691S) for 1 h at room temperature or overnight at 4°C on a rocking platform. After washing in PBST (0.1% Tween-20, PBS) three times, HRP conjugated secondary antibodies diluted in 0.5% skimmed milk in PBST were applied to the membrane for 10 min at room temperature on a rocking platform followed by four washes in PBST 20 min each. Washing and secondary antibodies steps were performed using SNAP id protein detection system (Sigma-Aldrich). Proteins were detected using Immobilon Western Chemiluminescent HRP substrate (Millipore WBLUC0500) or ECL blotting reagents (GE Healthcare GERPN2109).

Immune capillary electrophoresis

For protein AKT phosphorylation analysis, 3D cultures were starved in FAD medium 0.1% FCS without cholera toxin, epidermal growth factor, insulin and hydrocortisone for 16 h at 37°C 5% CO_2. Then treated for 15 min in the same starving medium or FAD medium with cholera toxin, epidermal growth factor, insulin, hydrocortisone and 20% FCS with or without LY294002 (50 µM). Cultures were lysed in ice-cold RIPA buffer (Thermo Scientific, 89900) containing protease and phosphatase inhibitors (Thermo Scientific, 78415). Plates were frozen at -80°C and thawed on ice, scraped and passed twice through a Qiashredder (2 min centrifuged at maximum speed), then incubated 1 h on ice vortexing every 15 min. Then lysates were centrifuged at 14000 g for 20 min at 4°C. The supernatant was collected for analysis. Total protein quantification was performed using Pierce BCA Protein Assay Kit (Thermo Scientific, 23227). For HIF1A analysis, 3D cultures were treated for 16h in starvation condition as for AKT analysis with or without LY294002 (0.5 µM). Then cultures were scraped on ice in ice-cold RIPA buffer (Thermo Scientific, UK) containing protease and phosphatase inhibitors and MG132 100uM proteasome inhibitor. Lysates were passed through a 23G needle 20 times and incubated for 15 min on ice. Next centrifuged at 14000G for 10min at 4ºC and the supernatant transferred to a pre-chilled new tube. Immune capillary electrophoresis was performed using Wes Simple™ (ProteinSimple, P/N 031-108) following manufacturer's instructions. Primary antibodies used were p-AktS493 (New England Biolabs 4060S, 1:150), Akt (New England Biolabs 4691S, 1:100), p-PRAS40 (New England Biolabs 2691T, 1:300), PRAS40 (New England Biolabs 2997T, 1:150), HIF1A (Novus Biologicals NB100-134, 1:50) and α-tubulin (New England Biolabs 2125S, 1:2000).

Western blotting

Induced or uninduced cultures were treated and collected as for the immune capillary electrophoresis. Protein was quantified with the Bio-Rad Protein Assay Kit (Bio-rad). Cell lysates were mixed with equal amount of 4x loading buffer (30 mM Tris-HCl pH 7, 7.5% SDS, 30% Glycerol, 0.05% Bromophenol blue and 400mM DTT) and boiled at 96°C for 5 min. Samples were loaded onto a 10% of SDS-polyacrylamide gel. Proteins were separated by electrophoresis using Bio-rad Mini-Protean II electrophoresis cell and transferred onto Nitrocellulose membrane (pore size 0.45 µm, Bio-rad 1620115). Membranes were incubated in blocking buffer (5% dried skimmed milk, TBS, 0.5% Tween-20) at room temperature for 1 h and then with primary antibodies diluted in blocking buffer (anti-P-AKTS473 Cell Signaling 4060S 1:2000; anti-pAKT308 Cell Signaling 2965S 1:1000; anti-Akt Cell Signaling 4691S 1:1000; anti-PGSK3β Cell Signaling 9322S 1:1000; anti-GSK3β Cell Signaling 9315S 1:1000; anti-P-S6 Cell Signaling 2211 1:1000, anti-alpha-tubulin Cell Signaling 2125S 1:1000) for 1 h at room temperature or overnight at 4°C on a rocking platform. After washing in TBST (0.5% Tween-20, TBS) three times, HRP conjugated secondary antibodies diluted in 0.5% skimmed milk in TBS were applied to the membrane for 45 min at room temperature on a rocking platform followed by three washes in TBST. Proteins were detected using ECL blotting reagents (Biological Industries 20-500-120).

CRISPR screening

Library cloning

Gene targets affecting the PI3K pathway, glycolysis, intracellular pH, and the HIF pathway were selected. The 20 essential genes most highly expressed in mouse esophageal primary epithelioid cultures were selected from the Achilles project essential genes[27]. For each target 4 gRNAs were selected from the Brie library [28] and 6 using the ChopChop tool v3 [29], selecting the best ranked ones. 100 non targeting gRNAs from Brie library were used as controls. Flanking sequences were added to make them compatible for Gibson assembly into the pKLV2-U6gRNA5(BbsI)-ccdB-PGKpuro2ABFP-W vector (gift from E. Metzakopian). Library was ordered as an ssDNA oligonucleotide pool (90bp each) from Twist Bioscience, USA. The pool was PCR amplified (2X Q5 HotStart, NEB) for 10 cycles (98 °C for 30s, 98 °C for 10s, 67 °C for 10s and 72 °C for 15 s, final extension of 72 °C for 2 min, infinite hold at 4 °C). Amplicons were PCR-cleaned, eluted (QIAquick PCR purification kit, Qiagen) and cloned into the backbone vector at 5:1 (vector:insert) ratio by Gibson assembly (GeneArt Gibson Assembly HiFi, ThermoFisher). The reaction product was used to transform Lucigen Endura™ ElectroCompetent Cells following manufacturer's instructions. 2 x 200ml LB flasks (supplemented with Ampicillin 100 ug/ml) were inoculated and grown at 37 °C for 16 h. Library DNA was extracted using a Qiagen EndoFree Plasmid Maxi Kit (Qiagen, 12362). DNA was stored at -20 °C until further use.

Lentivirus production

Lentiviral particles were produced by transfecting HEK293FT (ThermoFisher R70007) cells with packaging plasmids (psPax2 and pMD2.G, Addgene) and lentiviral library using Lipofectamine 3000 (ThermoFisher, L3000001). The following ratio was used for each transfection: 7.5µg psPax2, 2.5 µg pMD2.G and 6 µg sgRNA library per 10cm dish. Supernatant was collected 3 days post-transfection, filtered (Sartorius Minisart 0.45um) and stored at -80 °C until further use.

Cell culture

R26$^{Cas9\text{-}P2A\text{-}EGFP}$ mice (The Jackson Laboratory, 026179) were crossed with *Pik3ca*$^{fl\text{-}H1047R\text{-}T2A\text{-}EYFP\text{-}NLS/WT}$ mice. Primary epithelioid cultures established from *Pik3ca*$^{fl\text{-}H1047R\text{-}T2A\text{-}EYFP\text{-}NLS/WT}$;*R26*$^{Cas9\text{-}P2A\text{-}EGFP}$ mice were transduced with Ad-CMV-iCre or Ad-Null and maintained in culture for one week before the infection with the gRNA-library lentivirus. Two mice of the same genotype were used for each biological replicate and 2/3 replicates were performed in total for time 0 and two for time 3 weeks. The cells were incubated with 20% v/v lentivirus (~MOI 0.3) in the presence of 8 µg/µl Polybrene (Santa Cruz Biotechnology). Five days after the gRNA-library transduction, the cells pooled and ~ 2.5x10^{6} were collected for the 0 week time point. A fraction of the collected cells was used for FACS analysis (~2.5 x10^{6} cells) of the transduced cells and the rest were stored at -20°C as cell pellet until genomic DNA extraction. The remaining cells were seeded in a new well and cultured in minimal medium in the absence or presence of Insulin 5µg/ml until the collection of the final time point at 3 weeks.

Library preparation and sequencing

Illumina sequencing of the guide library and analysis of guide distribution was performed as follows. Guide library was PCR amplified by a two-step PCR. The first round of amplification (2X Q5 HotStart, NEB) was performed using the primers: gLibrary-HiSeq_50bp-SE-U1: ACACTCTTTCCCTACACGACGCTCTTCCGATCTCTTGTGGAAAGGACGAAACA; gLibrary-HiSeq_50bp-SE-L1: TCGGCATTCCTGCTGAACCGCTCTTCCGATCTCTAAAGCGCATGCTCCAGAC. PCR products were cleaned (QIAquick PCR purification kit, Qiagen) and diluted to 200pg/ul. 1ng was used as template to add indexing primers in 10 cycles. The amplicons were SPRI-bead purified (AMPure XP SPRI beads; Beckman Coulter), quantified (Qubit; Thermo Fisher Scientific) and quality checked (Bioanalyzer; Agilent). For gRNAs sequencing in CRISPR screen samples, genomic DNA was extracted with the DNAeasy Blood and Tissue kit (Qiagen). 3µg of DNA per sample was used for PCR amplification and indexing of the integrated gRNAs as described earlier. Libraries were sequenced on Illumina MiSeq by single-end sequencing for 20bp reads using a custom sequencing primer.

Data analysis

sgRNA counting was performed using an in-house script. Only perfect matches to the reference sequences were counted. All guides were detected in the plasmid library and initial time-points. Gini coefficients and Lorenz curves were calculated for all samples using the “Ineq” package in R and initial time-points and

plasmid library were verified as having even distributions. Gini coefficients for time 0 samples ranged between 0.169-0.171. Read-counts for each sample were normalized to reads per million fragments and corrected to account for differences in transduction efficiency between samples as reported by flow cytometry (Data in Supplementary Table 1). Enrichment analysis was done using the MAGeCK 0.5.9 software package [30]. Essential genes were analyzed separately. Samples were treated as unpaired, with the initial time-points being treated as controls (-c) and 3-week timepoints treated as test (-t). Non-targeting guides were defined as parameters using the --control-sgrna flag. Log fold changes per gene were provided by the MaGeCK gene summary output file. Log fold changes per replicate were calculated as the median Log Fold change of all gRNAs targeting the same gene in that replicate, average and standard deviation between the different replicates was calculated per condition to generate the plots in **Fig. 5c** and **Extended Data Fig. 5e**. Enrichment scores were calculated by summing the Log10 of the negative and positive enrichment scores provided by MAGeCK. Data was visualized using GraphPad[31].

Real-time qPCR

RNA extractions were performed on insert cultures as explained above. Total RNA was measured using Qubit™ RNA BR Assay Kit (Thermo Fishcer Scientific, catalog no. Q10211). cDNA synthesis of 500ng total RNA was performed using QuantiTect Reverse Transcription Kit (Qiagen, catalog no. 205313). RT-qPCR was performed with Taqman Fast Advanced Master Mix (Thermo Fisher Scientific, catalog no. 4444557) on StepOnePlus™ Real-Time PCR System (Thermo Fisher Scientific, catalog no. 4376600) and analyzed using StepOne Software v2.3. Relative q-PCR expression to Rplp0 housekeeping gene was calculated using delta-delta Ct method. The specific Taqman primers used for quantification were the following: Rplp0: Mm00725448_s1; HIF1α: Mm00468869_m1.

RNA isolation and RNA sequencing

Total RNA was extracted from 3D cultures of mouse primary keratinocytes after 1 week in FAD medium supplemented with fetal calf serum, apo-transferrin and Penicillin/Streptomycin, with or without insulin. RNA was extracted using RNeasy Micro Kit (QIAGEN, 74106), following the manufacturer's recommendations. Briefly, cells were washed with cold Hank's Balanced Salt Solution-HBSS (GIBCO, UK) and then lysis buffer was added directly to the insert. The integrity of total RNA was determined by Qubit RNA Assay Kit (Invitrogen, Q32852). For RNA-seq, libraries were prepared in an automated fashion using an Agilent Bravo robot with a KAPA Standard mRNA-Seq Kit (KAPA BIOSYSTEMS). In house adaptors were ligated to 100-300 bp fragments of dsDNA. All the samples were then subjected to 10 PCR cycles using sanger_168 tag set of primers and paired-end sequencing was performed on Illumina HiSeq 2500 with 75 bp read length. Reads were mapped using STAR 2.5.3a, the alignment files were sorted and duplicate-marked using Biobambam2 2.0.54, and the read summarization performed by the htseq-count script from version 0.6.1p1 of the HTSeq framework [32,33]. For GSEA analysis raw counts were normalized by the median of ratios method [34]. Gene set enrichment was analyzed with GSEA software [35] using the Hallmarks gene sets of the Molecular Signature Database (MSigDB) version 4.0 provided by the Broad Institute (http://www.broad.mit.edu/gsea/), following the standard procedure described on the GSEA user guide

(http://www.broadinstitute.org/gsea/doc/GSEAUserGuideFrame.html). The False Discovery Rate (FDR) for GSEA is the estimated probability that a gene set with a given NES (normalized enrichment score) represents a false-positive finding, and an FDR<0.25 is considered to be statistically significant for GSEA. FDR is adjusted for gene set size and multiple hypotheses testing. Nominal p-value is obtained using an empirical phenotype-based permutation test procedure and it is not adjusted for multiple comparisons[35].

Differential gene expression was analyzed using the DEBrowser tool (https://debrowser.umassmed.edu/) with which we performed a DESeq2 analysis [34] filtering the low counts to remove genes with less than 2 cpm in at least 2 samples. Parametric' fitting of dispersions to the mean intensity was used with the likelihood ratio test on the difference in deviance between a full and reduced model formula (defined by nbinomLRT). P-values obtained from Wald test are adjusted for multiple comparisons using the using the Benjamini and Hochberg method. An adjusted p-value cut-off of 0.05 were used to select significantly different expressed genes. Heatmaps were generated from the TPM values and build using ClustVis (https://biit.cs.ut.ee/clustvis/) and Morpheus tools (https://software.broadinstitute.org/morpheus/), significance was calculated from the adjusted p-value obtained in the DE analysis. Kyoto Encyclopedia of Genes and Genomes (KEGG) pathway enrichment analysis was performed uploading the significantly upregulated gene list (adjusted p<0.05) into the Enrichr tool (https://amp.pharm.mssm.edu/Enrichr/) [36]. Pathway p-values are adjusted for multiple hypotheses testing using the Benjamini-Hochberg method. Venn diagrams were generated using the Venn Diagrams tool (https://www.biotools.fr/misc/venny). MA plots were generated using GraphPad Prism 8.

Supplementary Figure 1

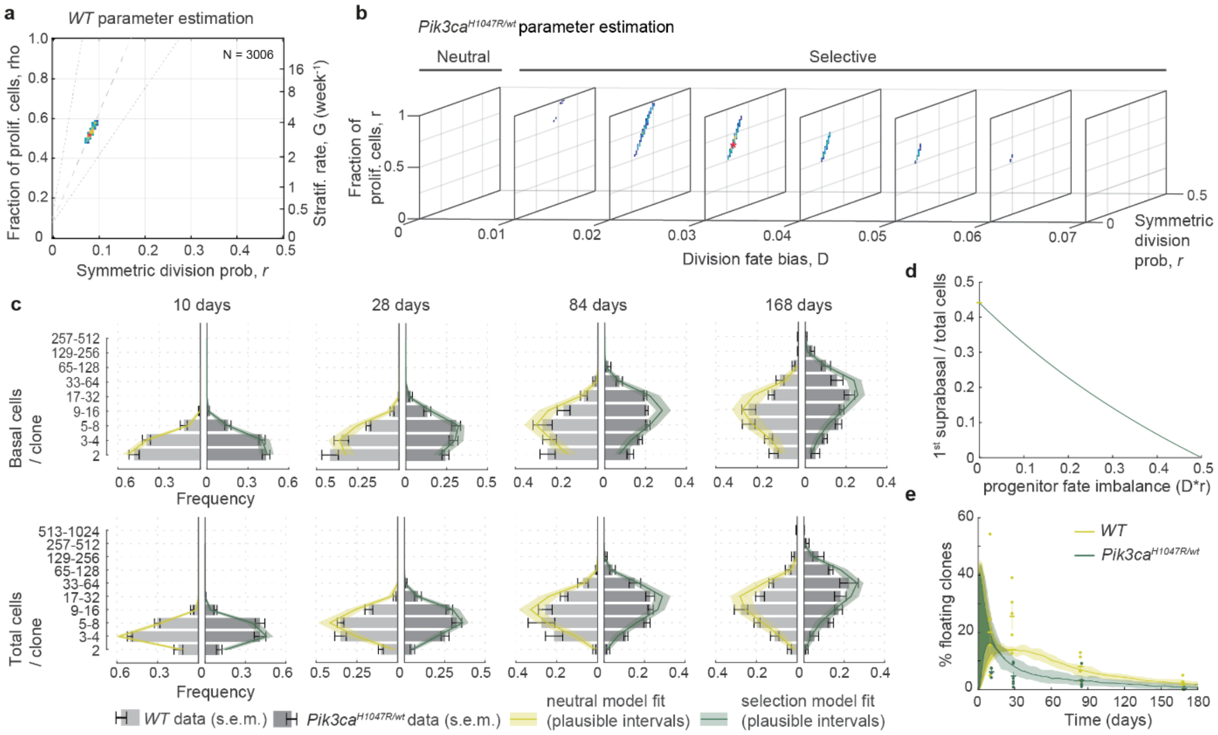

Mathematical modelling of clonal dynamics a-b, Parameter inference for wild-type (**a** and mutant (**b**) progenitor cells. Distributions of basal cells/clone were fitted (see **Supplementary Note**). Heatmaps show likeliest parameter values according to likelihood inference for a neutral single-progenitor model with balanced (wild-type, **a**) and with imbalanced fates (mutant, **b**). In the latter, different values for fate bias Δ were considered. Red asterisk: maximum likelihood estimate (MLE). Colored regions fall within 95% CI (uncolored regions are out of bounds). **c**, Distributions of wild-type (light grey, left) and mutant (dark grey, right) clone sizes from Figure 3d. Number of basal cells/clone, and number of total (basal + first suprabasal layer) cells/clone are displayed (top and bottom panels, respectively) (sizes grouped in powers of two). Error bars: experimental mean ± s.e.m. Overlaid are MLE model fits (shaded areas represent 95% plausible intervals given the total number of clones counted at each time point). **d**, Theoretical prediction of effect of progenitor fate imbalance on relative proportion of first suprabasal layer cells, **Supplementary Note,** initial departure point corresponds to wild-type value. **e**, Proportion of clones with 0 basal cells over time. Yellow and green dots represent means in individual wild-type and mutant mice, respectively (error bars: s.e.m.). Overlaid are MLE model fits once changes in suprabasal-to-total cell ratio over time were considered (shaded areas defined as in **c**). Source data are shown in Supplementary Table 1.

Supplementary Figure 2

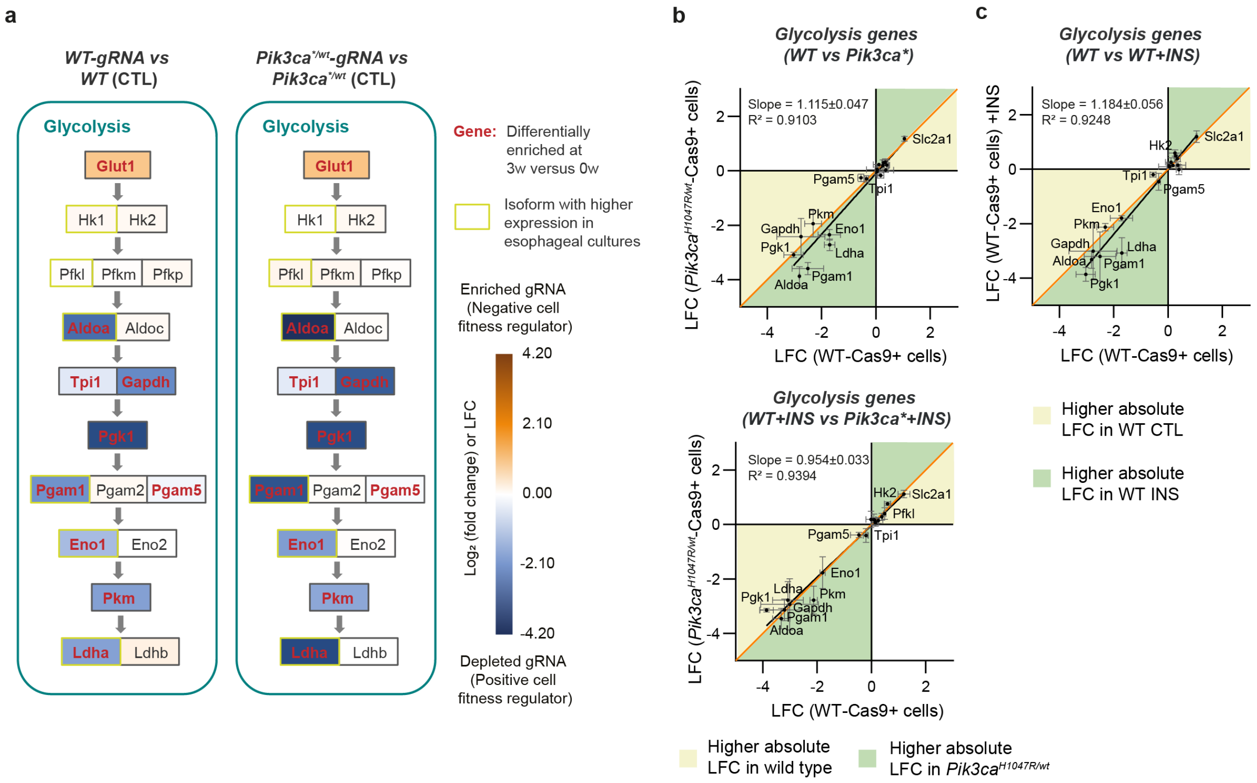

CRISPR screening results of glycolysis-related targets affecting mutant cell fitness. a, Illustration of gene targets related to the glycolysis pathway in CRSIPR screens performed in uninduced (WT, left panel) or induced (*Pik3ca*$^{*/wt}$, right panel) primary cells from the *Rosa26*$^{Cas9/wt}$ *Pik3ca*$^{H1047R/wt}$ mice. Box color indicates the Log_2 (fold change) between 3 and 0 weeks as indicated by color scale. Bold red indicates significantly enriched or depleted genes (FDR<0.1 and >10% Fold change). Yellow boxes: enzyme isoform most expressed in esophageal cells. **b-c**, Plot shows average Log_2 (fold change) of gRNA targeting indicated genes in the specified CRISPR screen conditions. Panels indicate gene-sets of Glycolysis pathway genes. b, CRISPR screens performed in *Pik3ca*$^{H1047R/wt}$ cells (y-axis) versus WT cells (x-axis) in control (CTL, top) or insulin-treated condition (INS, bottom). c, CRISPR screens performed in WT cells in CTL (x-axis) versus INS treated (y-axis). Yellow and green areas indicate higher absolute Log_2 (fold change) in wild-type or *Pik3ca*$^{H1047R/wt}$ cells respectively. Linear regression black with slope and coefficient of Determination R^2. Identity line, orange. Error bars, S.D., n=2-3 independent screens.

Supplementary Figure 3

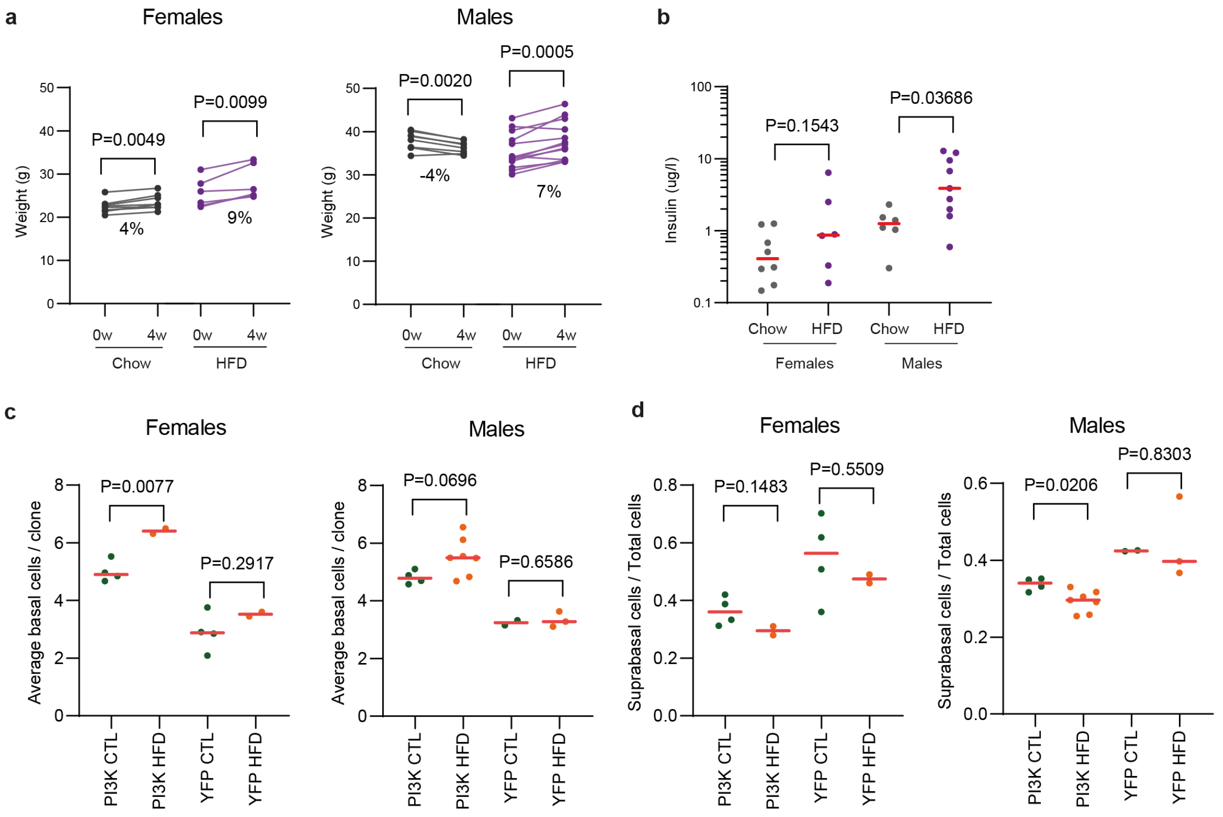

Sex-separated analysis of HFD effect on clone sizes. Mice were fed for 4 weeks with a normal chow or high-fat diet. **a,** Body weight measured at weeks 0 and 4 post diets classifying animals per gender. Each dot corresponds to one animal, lines link two weights of the same animal (n=10-16 mice). Two-tailed paired *t*-test. **b**, Insulin levels in blood, measured at the end of the experiment in animals from a. Two-tailed unpaired *t*-test. **c-d**, Wild-type (*Cre-RYFP, YFP)* and mutant (*Cre-Pik3ca*$^{H1047R\text{-}YFP/wt}$, PI3K) mice were induced fed a normal chow (CTL) or high fat diet (HFD) and tissues collected 28 days post-induction. **c**, Average basal clone sizes for each strain and treatment and sex, considering all clones with at least one basal cell. Dots indicate the average clone size of a mouse. n=5-9 mice. Two-tailed unpaired *t*-test. Source data are shown in Supplementary Table 1. **d**, Average proportion of suprabasal cells per clone for each strain and treatment classifying animals per gender, only first suprabasal cells were counted. Each dot corresponds to one animal. n=5-9 mice. Two-tailed unpaired *t*-test.

Supplementary Note References

1. Banreti, A.R. & Meier, P. The NMDA receptor regulates competition of epithelial cells in the Drosophila wing. *Nature Communications* **11**, 2228 (2020).
2. de la Cova, C. *et al.* Supercompetitor status of Drosophila Myc cells requires p53 as a fitness sensor to reprogram metabolism and promote viability. *Cell Metab* **19**, 470-83 (2014).
3. Hamanaka, R.B. & Mutlu, G.M. PFKFB3, a Direct Target of p63, Is Required for Proliferation and Inhibits Differentiation in Epidermal Keratinocytes. *J Invest Dermatol* **137**, 1267-1276 (2017).
4. Sutter, C.H., Olesen, K.M., Bhuju, J., Guo, Z. & Sutter, T.R. AHR Regulates Metabolic Reprogramming to Promote SIRT1-Dependent Keratinocyte Differentiation. *J Invest Dermatol* **139**, 818-826 (2019).
5. Cliff, T.S. *et al.* MYC Controls Human Pluripotent Stem Cell Fate Decisions through Regulation of Metabolic Flux. *Cell Stem Cell* **21**, 502-516.e9 (2017).
6. Miao, W. *et al.* Glucose dissociates DDX21 dimers to regulate mRNA splicing and tissue differentiation. *Cell* **186**, 80-97.e26 (2023).
7. Hamanaka, R.B. & Mutlu, G.M. PFKFB3, a Direct Target of p63, Is Required for Proliferation and Inhibits Differentiation in Epidermal Keratinocytes. *Journal of Investigative Dermatology* **137**, 1267-1276 (2017).
8. Michelakis, E.D., Webster, L. & Mackey, J.R. Dichloroacetate (DCA) as a potential metabolic-targeting therapy for cancer. *Br J Cancer* **99**, 989-94 (2008).
9. James, M.O. *et al.* Therapeutic applications of dichloroacetate and the role of glutathione transferase zeta-1. *Pharmacol Ther* **170**, 166-180 (2017).
10. Lingohr, M.K., Thrall, B.D. & Bull, R.J. Effects of Dichloroacetate (DCA) on Serum Insulin Levels and Insulin-Controlled Signaling Proteins in Livers of Male B6C3F1 Mice. *Toxicological Sciences* **59**, 178-184 (2001).
11. Yang, L., Venneti, S. & Nagrath, D. Glutaminolysis: A Hallmark of Cancer Metabolism. *Annual Review of Biomedical Engineering* **19**, 163-194 (2017).
12. Koundouros, N. & Poulogiannis, G. Reprogramming of fatty acid metabolism in cancer. *British Journal of Cancer* **122**, 4-22 (2020).
13. Hoxhaj, G. & Manning, B.D. The PI3K-AKT network at the interface of oncogenic signalling and cancer metabolism. *Nat Rev Cancer* **20**, 74-88 (2020).
14. Ricoult, S.J.H., Yecies, J.L., Ben-Sahra, I. & Manning, B.D. Oncogenic PI3K and K-Ras stimulate de novo lipid synthesis through mTORC1 and SREBP. *Oncogene* **35**, 1250-1260 (2016).
15. Lau, C.E., Tredwell, G.D., Ellis, J.K., Lam, E.W. & Keun, H.C. Metabolomic characterisation of the effects of oncogenic PIK3CA transformation in a breast epithelial cell line. *Sci Rep* **7**, 46079 (2017).
16. Doupe, D.P. *et al.* A single progenitor population switches behavior to maintain and repair esophageal epithelium. *Science* **337**, 1091-3 (2012).
17. Piedrafita, G. *et al.* A single-progenitor model as the unifying paradigm of epidermal and esophageal epithelial maintenance in mice. *Nature Communications* **11**, 1429 (2020).
18. Clayton, E. *et al.* A single type of progenitor cell maintains normal epidermis. *Nature* **446**, 185-189 (2007).
19. Murai, K. *et al.* Epidermal Tissue Adapts to Restrain Progenitors Carrying Clonal p53 Mutations. *Cell Stem Cell* **23**, 687-699.e8 (2018).
20. Gillespie, D. Exact Stochastic Simulation of Coupled Chemical Reactions. *The Journal of Physical Chemistry* **81**, 2340-2361 (1977).
21. Gillespie, D.T. A general method for numerically simulating the stochastic evolution of coupled chemical reactions. *Journal of Computational Physics* **22**, 403-434 (1976).
22. Fernandez-Antoran, D. *et al.* Outcompeting p53-Mutant Cells in the Normal Esophagus by Redox Manipulation. *Cell Stem Cell* **25**, 329-341 (2019).

23. Alcolea, M.P. *et al.* Differentiation imbalance in single oesophageal progenitor cells causes clonal immortalization and field change. *Nat Cell Biol* **16**, 615-22 (2014).
24. Klein, A.M., Brash, D.E., Jones, P.H. & Simons, B.D. Stochastic fate of *p53*-mutant epidermal progenitor cells is tilted toward proliferation by UV B during preneoplasia. *Proceedings of the National Academy of Sciences* **107**, 270-275 (2010).
25. Colom, B. *et al.* Spatial competition shapes the dynamic mutational landscape of normal esophageal epithelium. *Nature Genetics* **52**, 604–614 (2020).
26. Liu, N. *et al.* Stem cell competition orchestrates skin homeostasis and ageing. *Nature* **568**, 344-350 (2019).
27. Dempster, J.M. *et al.* Extracting Biological Insights from the Project Achilles Genome-Scale CRISPR Screens in Cancer Cell Lines. *bioRxiv*, 720243 (2019).
28. Doench, J.G. *et al.* Optimized sgRNA design to maximize activity and minimize off-target effects of CRISPR-Cas9. *Nature Biotechnology* **34**, 184-191 (2016).
29. Labun, K. *et al.* CHOPCHOP v3: expanding the CRISPR web toolbox beyond genome editing. *Nucleic Acids Res* **47**, W171-w174 (2019).
30. Li, W. *et al.* MAGeCK enables robust identification of essential genes from genome-scale CRISPR/Cas9 knockout screens. *Genome Biology* **15**, 554 (2014).
31. Wang, B. *et al.* Integrative analysis of pooled CRISPR genetic screens using MAGeCKFlute. *Nature Protocols* **14**, 756-780 (2019).
32. Dobin, A. *et al.* STAR: ultrafast universal RNA-seq aligner. *Bioinformatics* **29**, 15-21 (2013).
33. Anders, S., Pyl, P.T. & Huber, W. HTSeq--a Python framework to work with high-throughput sequencing data. *Bioinformatics* **31**, 166-9 (2015).
34. Love, M.I., Huber, W. & Anders, S. Moderated estimation of fold change and dispersion for RNA-seq data with DESeq2. *Genome Biol* **15**, 550 (2014).
35. Subramanian, A. *et al.* Gene set enrichment analysis: a knowledge-based approach for interpreting genome-wide expression profiles. *Proc Natl Acad Sci U S A* **102**, 15545-50 (2005).
36. Kuleshov, M.V. *et al.* Enrichr: a comprehensive gene set enrichment analysis web server 2016 update. *Nucleic Acids Res* **44**, W90-7 (2016).
