# Supplementary material for: Organismal metabolism regulates the expansion of oncogenic PIK3CA mutant clones in normal esophagus
Source: Nat Genet. 2024 Aug 21;56(10):2144–57. doi: 10.1038/s41588-024-01891-8 (PMC11525199; doi:10.1038/s41588-024-01891-8)
Supplement: Supplementary file 2 — Reporting Summary [file 41588_2024_1891_MOESM2_ESM.pdf]

nature portfolio

Corresponding author(s): Philip H Jones

Last updated by author(s): Jul 12, 2024

Reporting Summary

Nature Portfolio wishes to improve the reproducibility of the work that we publish. This form provides structure for consistency and transparency in reporting. For further information on Nature Portfolio policies, see our Editorial Policies and the Editorial Policy Checklist.

Statistics

For all statistical analyses, confirm that the following items are present in the figure legend, table legend, main text, or Methods section.

n/a	Confirmed	
☐	☒	The exact sample size (*n*) for each experimental group/condition, given as a discrete number and unit of measurement
☐	☒	A statement on whether measurements were taken from distinct samples or whether the same sample was measured repeatedly
☐	☒	The statistical test(s) used AND whether they are one- or two-sided<br>*Only common tests should be described solely by name; describe more complex techniques in the Methods section.*
☐	☒	A description of all covariates tested
☐	☒	A description of any assumptions or corrections, such as tests of normality and adjustment for multiple comparisons
☐	☒	A full description of the statistical parameters including central tendency (e.g. means) or other basic estimates (e.g. regression coefficient) AND variation (e.g. standard deviation) or associated estimates of uncertainty (e.g. confidence intervals)
☐	☒	For null hypothesis testing, the test statistic (e.g. *F*, *t*, *r*) with confidence intervals, effect sizes, degrees of freedom and *P* value noted<br>*Give P values as exact values whenever suitable.*
☐	☒	For Bayesian analysis, information on the choice of priors and Markov chain Monte Carlo settings
☒	☐	For hierarchical and complex designs, identification of the appropriate level for tests and full reporting of outcomes
☐	☒	Estimates of effect sizes (e.g. Cohen's *d*, Pearson's *r*), indicating how they were calculated

Our web collection on statistics for biologists contains articles on many of the points above.

Software and code

Policy information about availability of computer code

Data collection	Confocal images were obtained using the Leica Adquisiton Software LAS X. Flow cytometry data was obtained using a BD LSRFortessa and BD FACSDiva•M Software (BO-Biosciences). Immune capillary electrophoresis was performed using Wes Simple'M (ProteinSimple, P/N 031-108) with the Compass software. OCR and ECAR were assayed using the XF24 software in a Seahorse XF-24 analyser. DNA sequencing was performed on an lllumina MiSeq platform. RNA sequencing was performed on an lllumina HiSeq 2500 platform.
Data analysis	- Confocal Z stack images were rendered and analyzed with lmaris 4.3 (Bitplane), ImageJ software, or Volocity 6.3 Software (Perkin Elmer).<br>- Flow cytometry data was analysed using the FlowJo software (version 10.5.3).<br>- Immune capillary electrophoresis analysis was performed using the Compass software.<br>- OCR and ECAR were analysed using the XF24 software.<br>- RNA sequencing: Reads were mapped using STAR 2.5.3a; the alignment files were sorted and duplicate-marked using Biobambam2 2.0.54; the read summarization performed by the htseq-count script from version 0.6.lpl of the HTSeq framework; Gene set enrichment was analyzed with GSEA software, using the Hallmarks gene sets of the Molecular Signature Database (MSigDB) version 4.0 provided by the Broad Institute (http://www.broad.mit.edu/gsea/), following the standard procedure described on the GSEA user guide (http:// www.broadinstitute.org/gsea/doc/GSEAUserGuideFrame.html); Differential gene expression was analyzed using the DEBrowser tool (https:// debrowser.umassmed.edu/); Heatmaps were generated from the TPM values and build using ClustVis (https://biit.cs.ut.ee/clustvis/) and Morpheus tools (https:/ /software.broadinstitute.org/morpheus/); Kyoto Encyclopedia of Genes and Genomes (KEGG) pathway enrichment analysis was performed uploading the significantly upregulated gene list (p<0.05) into the Enrichr tool (https://amp.pharm.mssm.edu/ Enrichr/); Venn diagrams were generated using the Venn Diagrams tool (https:/ /www.biotools.fr/misc/venny); MA plots were generated using GraphPad Prism 8v8.3.1.<br>- DNA sequencing analysis: ShearwaterML algorithm from the deepSNV package (vl.21.3, https://github.com/gerstung-lab/deepSNV), ClinVar mutation database (https:/ /clinvarminer.genetics.utah.edu/variants-by-gene/PIK3CA), Clinical Knowledgebase (https:/ /ckb.jax.org/gene/

- Lineage tracing: Code used for modelling has been made publicly available and can be found at https://github.com/gpl0/DriverClonALTfate. Code used to generate two-dimensional histograms of clone sizes, displayed as heatmaps is available in the CloneSizeFreq_2Dheat package (https ://githu b.com/gp 10/CloneSizeFreq_2 Dheat).
- Crispr screen: targets were selected using the ChopChop tool v3; Gini coefficients and Lorenz curves were calculated for all samples using the "lneq" package in R; Enrichment analysis was done using the MAGeCK 0.5.9 software package; Enrichment scores were further analysed using the MAGeCKFLUTE package) and visualised using Graph Pad.
- Statistics: GraphPad Prism software v8.3.l, Matlab, ARTool [R] package vignette, Matlab, CloneSizeFreq_2Dheat package (https:// github.com/gp10/CloneSizeFreq_2Dheat.

For manuscripts utilizing custom algorithms or software that are central to the research but not yet described in published literature, software must be made available to editors and reviewers. We strongly encourage code deposition in a community repository (e.g. GitHub). See the Nature Portfolio guidelines for submitting code & software for further information.

Data

Policy information about availability of data

All manuscripts must include a data availability statement. This statement should provide the following information, where applicable:

- Accession codes, unique identifiers, or web links for publicly available datasets
- A description of any restrictions on data availability
- For clinical datasets or third party data, please ensure that the statement adheres to our policy

The sequencing data sets in this study are publicly available at the European Nucleotide archive (ENA) Accession numbers for RNAseq data on https:// www.ebi.ac.uk/ena are as follows: In vivo samples: ERS14340821, ERS14340822, ERS14340823, ERS14340824. In vitro samples: ERS2515249, ERS2515250, ERS2515251, ERS2515252. Accession numbers for targeted DNA sequencing of SCA is ERP107379. Data used to generate each figure is available in Supplementary table 1.

Human research participants

Policy information about studies involving human research participants and Sex and Gender in Research.

Reporting on sex and gender	Our study used human samples from both sexes.
Population characteristics	Deceased organ donors from whom organs were being retrieved for transplantation. The samples a small unselected sample of organ donors in the Eastern region of England.
Recruitment	Written informed consent was obtained from relatives of deceased organ donors from whom organs were being retrieved for transplantation
Ethics oversight	Ethical approval was obtained from the Cambridge South Ethics Committee, Research Ethics Committee reference: 15/ EE/0152 NRES Committee East of England - Cambridge South

Note that full information on the approval of the study protocol must also be provided in the manuscript.

Field-specific reporting

Please select the one below that is the best fit for your research. If you are not sure, read the appropriate sections before making your selection.

☒ Life sciences ☐ Behavioural & social sciences ☐ Ecological, evolutionary & environmental sciences

For a reference copy of the document with all sections, see nature.com/documents/nr-reporting-summary-flat.pdf

Life sciences study design

All studies must disclose on these points even when the disclosure is negative.

Sample size	Sample size was not predetermined by statistical methods. Sample size for for lineage tracing and carcinogenesis studies was determined by previous studies (PMID: 22821983, PMID: 24814514, PMID: 31327664, PMID: 27548914, PMID: 34646013, PMID: 36266286 and PMID: 32424351).
Data exclusions	No data was excluded from the study.
Replication	Different epithelioid cultures or mice were considered as independent experimental unit. All attempts at replication were successful.
Randomization	Cultures and mice were randomly allocated in experimental groups.
Blinding	Investigators were not blinded as to sample IDs during analyses. The staining pattern of mutant clones and the evolving clone size distributions reveal the genotype and the time point, preventing effective blinding.

Reporting for specific materials, systems and methods

We require information from authors about some types of materials, experimental systems and methods used in many studies. Here, indicate whether each material, system or method listed is relevant to your study. If you are not sure if a list item applies to your research, read the appropriate section before selecting a response.

Materials & experimental systems

n/a	Involved in the study	
☐	☒	Antibodies
☐	☒	Eukaryotic cell lines
☒	☐	Palaeontology and archaeology
☐	☒	Animals and other organisms
☒	☐	Clinical data
☒	☐	Dual use research of concern

Methods

n/a	Involved in the study	
☒	☐	ChIP-seq
☐	☒	Flow cytometry
☒	☐	MRI-based neuroimaging

Antibodies

Antibodies used

Microscopy/Flow Cytometry

GFP	Life technologies	A10262	Polyclonal
Caspase 3	Abcam	ab2302	Polyclonal
Vimentin	Abcam	ab92457	EPR3776
ITGA6	Biolegend	313610	GoH3
Alexa Fluor 488	Jackson	703-545-155	
Donkey Anti-Chicken	ImmunoResearch		

Western Blotting/Protein Simple

P-Akt S473	CST	4060S	D9E
Akt	CST	4691S	C67E7
P-Akt T308	CST	2965S	C31E5E
PRAS40	CST	2691T	C77D7
P-PRAS40	CST	2997T	D23C7
HIF1A	Novus Biologicals	NB100-134	Polyclonal
GSK3b	CST	9315S	27C10
PGSK3b	CST	9322S	D3A4
PS6	CST	2211	Polyclonal
p110a	Abcam	Ab152155	Polyclonal
GFP	Abcam	Ab290	Polyclonal
aTubulin	CST	2125S	11H10

Validation

GFP: Detects GFP with no cross reactivity with mammalian proteins, data on manufacturer's website.
Caspase 3: staining induced by irradiation of mouse esophagus (PMC6739485)
ITGA6: Staining undetectable in knockout mice (PMID: 8673141)
Vimentin: Knockout validated, Abcam website
P-Akt S473: Validation by inhibitors, CST Website
Akt: Western blot in multiple cell lines and vs recombinant proteins CST Website
P-Akt T308: Validation by inhibitors, CST Website
PRAS40: siRNA knockdown abolishes WB band PMID: 23460019
P-PRAS40: Validation by inhibitors, CST Website
HIF1A: siRNA knockdown, this paper.
GSK3b: Data on CST Website.
PGSK3b: Data on CST Website.
PS6: WB and inhibitor validation CST Website
aTubulin: Data on CST Website.
p110a: Product correct size on WB, Ab no longer available.

Eukaryotic cell lines

Policy information about cell lines and Sex and Gender in Research

Cell line source(s): NIH 3T3 Cell line (ATCC CRL-1658)

Authentication	None of the cell lines used were authenticated
Mycoplasma contamination	The cells tested negative for mycoplasma.
Commonly misidentified lines (See ICLAC register)	No commonly misidentified cell lines were included in the study.

Animals and other research organisms

Policy information about studies involving animals; ARRIVE guidelines recommended for reporting animal research, and Sex and Gender in Research

Laboratory animals	All mouse strains were maintained on a C57/BL6 genetic background.<br>Strains used included:<br>Cyp1A1creERT<br>Rosa26FlYFP<br>Ins2Akita/wt<br>Pik3caflH1047RT2AYFP-NLS/wt<br>Rosa26Cas9P2AGFP<br>Rosa26FllConfetti<br>and crosses of the above lines.
Wild animals	The study did not involve wild animals.
Reporting on sex	Cultures were stablished from animals of both sexes. No sex specific differences were observed.
Field-collected samples	The study did not involve samples collected from the field.
Ethics oversight	All mouse experiments were ethically reviewed and approved by the Welcome Sanger Institute Ethics Committee and conducted according to UK government Home Office project licences PPL22/2282, PPL70/7543, and PPL4639B40.

Note that full information on the approval of the study protocol must also be provided in the manuscript.

Flow Cytometry

Plots

Confirm that:

☒ The axis labels state the marker and fluorochrome used (e.g. CD4-FITC).

☒ The axis scales are clearly visible. Include numbers along axes only for bottom left plot of group (a 'group' is an analysis of identical markers).

☒ All plots are contour plots with outliers or pseudocolor plots.

☒ A numerical value for number of cells or percentage (with statistics) is provided.

Methodology

Sample preparation	Primary cells grown as epithelioids were trypsinized to obtain a cell suspension.
Instrument	Becton Dickinson (BD) LSRFortessa
Software	FACSDiva™ Software (BD-Biosciences)<br>FlowJo software (version 10.5.3)
Cell population abundance	20000 single cells were analysed per sample.
Gating strategy	Single cells were selected using FSC-A/FSC-H and the cells expressing the fluorescent reporter quantified. YFP fluorescence was collected using the 488 nm laser and the 530/30 bandpass filter. ITGA6-647 fluorescence, to discriminate between basal and suprabasal cells, was collected using the 640 nm laser and the 670/14 bandpass filter.

☒ Tick this box to confirm that a figure exemplifying the gating strategy is provided in the Supplementary Information.
