# Supplementary material for: Organismal metabolism regulates the expansion of oncogenic PIK3CA mutant clones in normal esophagus
Source: Nat Genet. 2024 Aug 21;56(10):2144–57. doi: 10.1038/s41588-024-01891-8 (PMC11525199; doi:10.1038/s41588-024-01891-8)

Blots corresponding to Extended Data Figure 1c (top panel):

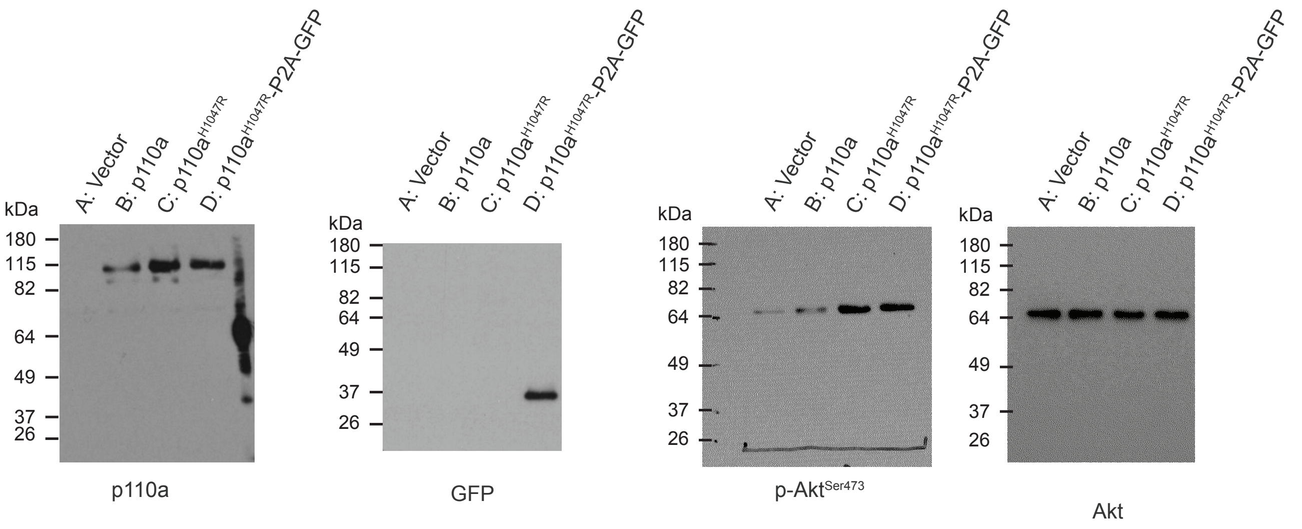


Blots corresponding to Extended Data Figure 1c (bottom panel):

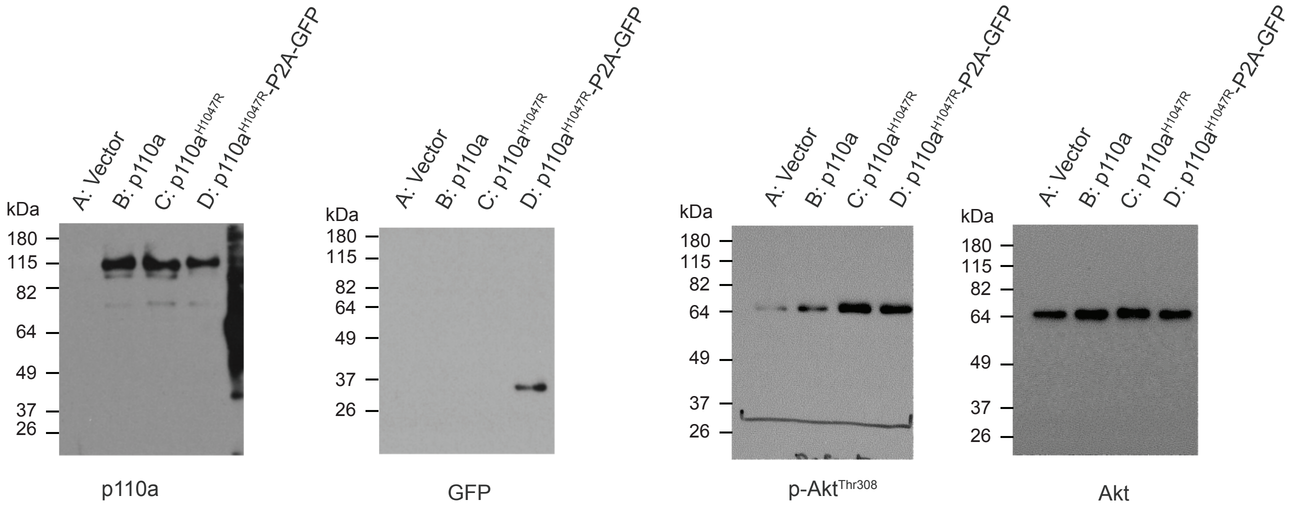


Blots corresponding to Figure 4e:

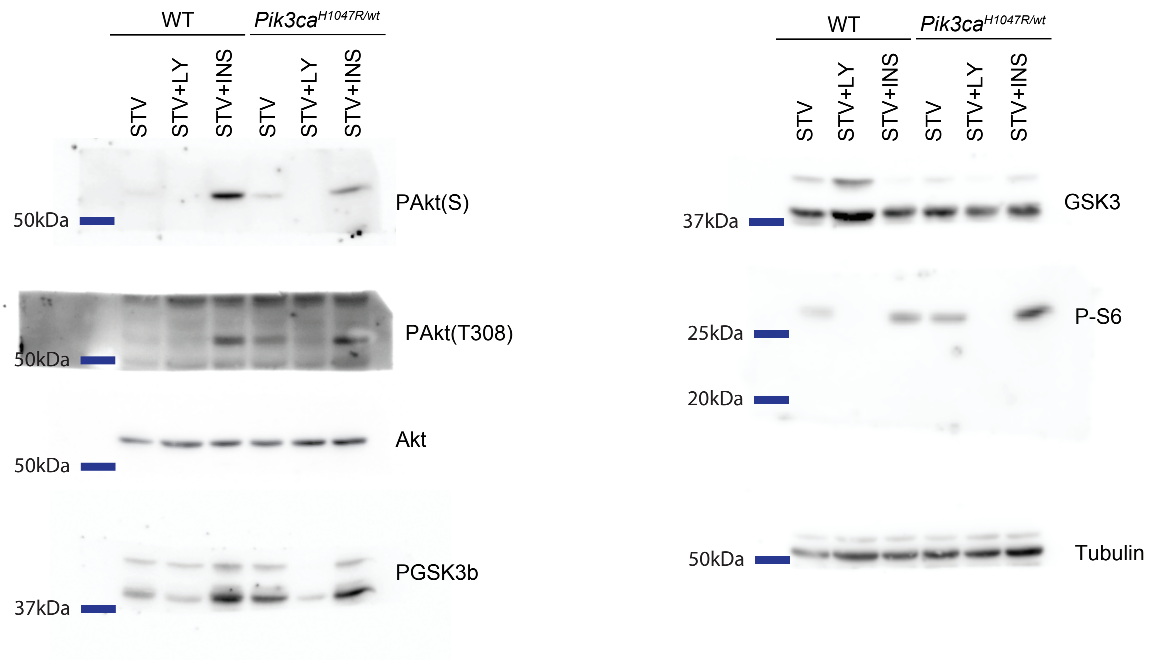

Supplement: Supplementary file 23 — Source data: scans of uncropped western blots. [file 41588_2024_1891_MOESM23_ESM.pdf]
